# Tape Transfer Atomization Patterning of Liquid Alloys for Microfluidic Stretchable Wireless Power Transfer

**DOI:** 10.1038/srep08419

**Published:** 2015-02-12

**Authors:** Seung Hee Jeong, Klas Hjort, Zhigang Wu

**Affiliations:** 1 Department of Engineering Sciences, The Angstrom Laboratory, Uppsala University, Box 534, 75121, Uppsala, Sweden; 2 State Key Laboratory of Digital Manufacturing Equipment and Technology, Huangzhong University of Science and Technology, 430074, Wuhan, China

## Abstract

Stretchable electronics offers unsurpassed mechanical compliance on complex or soft surfaces like the human skin and organs. To fully exploit this great advantage, an autonomous system with a self-powered energy source has been sought for. Here, we present a new technology to pattern liquid alloys on soft substrates, targeting at fabrication of a hybrid-integrated power source in microfluidic stretchable electronics. By atomized spraying of a liquid alloy onto a soft surface with a tape transferred adhesive mask, a universal fabrication process is provided for high quality patterns of liquid conductors in a meter scale. With the developed multilayer fabrication technique, a microfluidic stretchable wireless power transfer device with an integrated LED was demonstrated, which could survive cycling between 0% and 25% strain over 1,000 times.

In contrast to the traditional rigid and flexible electronics, stretchable electronics with a wireless communication interface offers unsurpassed mechanical compliance on human skin and organs^1^,^2^,^3^,^4^,^5^. Recently, *Science* presented a smart sensor system on the skin that was produced by hybrid assembly and liquid encapsulation^6^. This is a good complement to the silicon on insulator (SOI) based integrated circuits of the elastic electronics^5^. However, by adapting the same transfer process of thin film interconnects, it suffers from some of the disadvantages that large area elastic electronics has; high costs and high resistance interconnects, which induce low quality waveguides and antennas for large area microwave and radio frequency electronics.

In principle, liquids in elastic microchannels can reach an extreme level of deformation without noticeable hysteresis or permanent deformations since the liquids can flow without any discontinuity or mechanical resistance. Introducing conductive liquid patterns into soft materials, microfluidic stretchable electronics brings a new opportunity to fabricate stretchable electronics with thick and low-resistant conductors as well as passive devices^7^. Advances in microfluidic stretchable electronics have demonstrated quite a few interesting devices and applications. Using eutectic gallium based liquid alloys in polydimethylsiloxane (PDMS), a direct current (DC) circuit interconnector was first demonstrated^8^ with Indalloy 608 while we demonstrated the first high performance microfluidic radio frequency (RF) antenna using Galinstan^9^,^10^, and similar approaches have later been implemented for antennas with the eutectic gallium indium alloy, EGaIn^11^,^12^. Recently, we showed the potential in making pneumatically controllable 3D objects with a planar fabrication technique, by demonstrating a pneumatically tunable, hemispherical, small antenna^13^. By using the concept of localized stiff cells^14^, flexible circuitries with active components were integrated into large-area, single-layer^14^ and later multi-layer^15^ microfluidic devices to make a standalone functional electronic device or system for wireless sensor and communication. In addition, microfluidics based approaches that should be suitable for stretchable electronics have been studied for potential use in non-linear components such as tunnel junctions^16^ and memristors^17^, as well as electrochemical energy handling components for energy harvesting^18^ and storage^19^,^20^. Unfortunately, the fabrication techniques used for these devices are in a laborious handicraft stage, which is far from a manufacturing process for potential applications, such as wearable computing, healthcare or fitness monitoring, medical diagnostics, soft machines or soft robotics. Hence, the development of new batch processing and printing techniques has recently attracted high attention in academia^21^,^22^,^23^,^24^. In particular, batch processing has been demonstrated by liquid alloy printing with stencils^21^ or by masked deposition and selective wetting after dissolving the masked areas prior to depositing the metal^22^. However, due to the huge mismatch between the low surface energy of the common elastic substrate PDMS and the very high surface energy of the liquid alloy, these two liquid printing approaches demonstrated rough lines, which should severely reduce the electrical performance and especially so with line lengths in the scale of a meter or more. Recently, the possibility of atomized spraying of liquid alloys was demonstrated^25^,^26^.

Here, we propose a simple new liquid alloy patterning technique for microfluidic electronics by atomizing liquid alloys (with nitrogen gas as a carrier) and spraying them onto a semi-cured PDMS surface with a tape transferred adhesive mask, followed by encapsulation with another layer of an uncured PDMS. As mentioned earlier, some of the most attractive applications of stretchable electronics would be with compliant autonomous wireless sensor systems. Like any other portable devices or systems, the bottleneck is the power source for the systems, e.g., energy storage devices such as batteries and supercapacitors, or energy harvesters like solar cells, thermoelectric generators and piezoelectric generators. As one of the feasible power sources as itself and also a charging source of batteries or capacitors for stand-alone stretchable electronic systems, a stretchable long coil antenna for wireless power transfer is to be fabricated and evaluated with strain. Further with a multilayer processing and hybrid integration of rigid components, a microfluidic stretchable wireless power transfer device with a light emitting diode (LED) as an indicator is demonstrated, which could survive cycling strain between 0% and 25% over 1,000 times.

## Results

### Fabrication process with atomization patterning of liquid alloys

**Figure 1:**
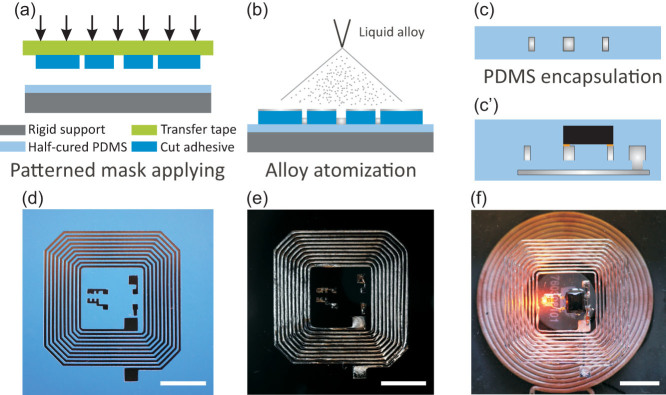
Schematic illustration of the critical processing steps (a)–(c) and the optical photos of samples in this processing (d)–(f). Transferring the cut vinyl patterned mask on a soft substrate(a), liquid alloy atomization patterning on the masked substrate (b), removing the mask (c) or alternatively mounting active components or a circuit (c′) followed by encapsulation, the photo of cut vinyl patterned mask on a PDMS substrate with a silicon wafer as support (d), the photo of a microfluidic induction coil (without active components) (e), and the photo of a microfluidic wireless power transfer device with active components inside (f), the scale bars indicate 10 mm (d–f).

Figure 1 (a–c) illustrates the core concept of the proposed fabrication process in this work, both for liquid alloy patterning and further rigid components (circuitry) hybrid integration. With tape transfer atomization patterning and the corresponding cut tape mask, Fig. 1 (d), a stretchable long coil antenna for a wireless power transfer device was shown in Fig. 1 (e). Further with a multilayer fabrication technique and hybrid integration, a stretchable wireless power transfer device with a hybrid integrated flexible circuit and an LED was for the first time demonstrated, Fig. 1 (f). A more detailed fabrication process can be found in Fig. S1 in the electronic supplementary information (ESI).

**Figure 2:**
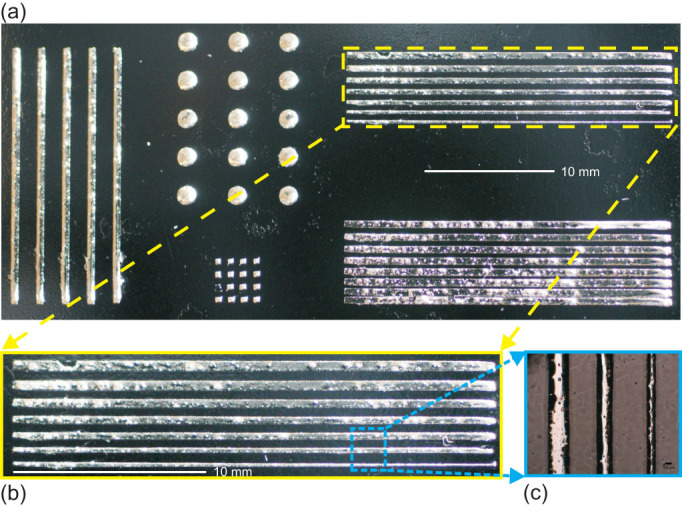
Micrographs of the patterned liquid alloys. The liquid alloy patterns on a PDMS substrate with a silicon wafer as support: various test patterns with different sizes and densities (a), and a zoomed photo on the lines with various widths (b)–(c).

**Figure 3:**
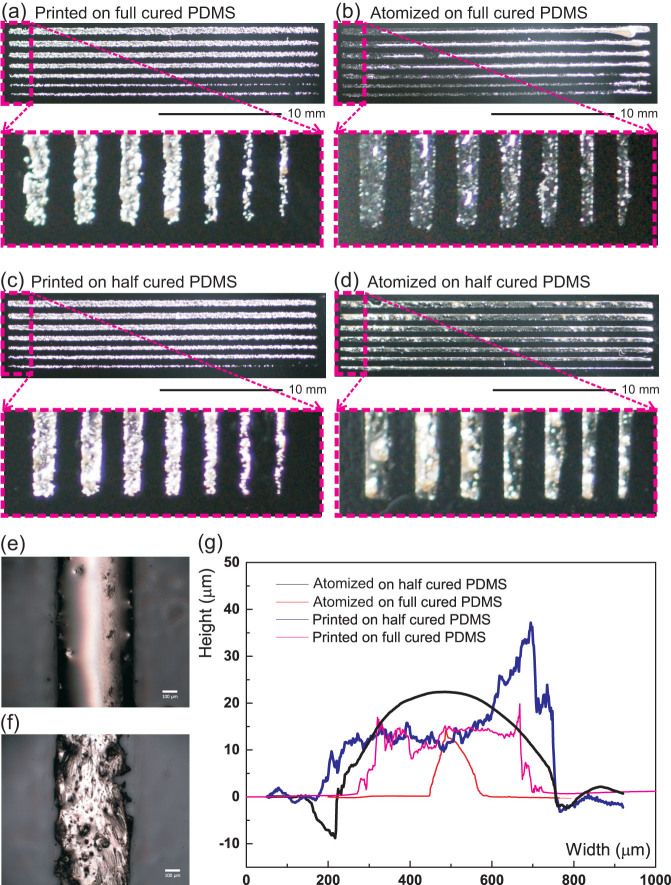
Surface comparison for liquid alloy patterning. Photos of printed (a) and atomization patterned (b) lines of liquid alloys which are 25 mm long and from 200 μm to 500 μm wide on a full cured PDMS and their counterparts (c)–(d) on a semi-cured PDMS substrate with a silicon wafer as support. Micrographs of representative patterned lines by atomization patterning (e) and liquid alloy printing (f) techniques and their morphology in height by an optical profiler (g).

Figure 2 shows optical micrographs of various lines and dot patterns of atomized liquid alloy patterns on a PDMS substrate with a 4-inch silicon wafer as support. In Fig. 2 (c), a precisely defined liquid alloy line by atomized spray deposition was shown with the smallest line width as 200 μm. Optical microscopy and profilometry show the shape definition and edge shape of liquid alloy patterns by liquid alloy printing and by atomization patterning on a fully cured and a semi-cured PDMS were studied, Fig. 3. The smoother and sharper shape of the atomized pattern on a semi-cured PDMS is shown in Fig. 3 (e)–(g). The tape transfer atomization patterned line on a semi-cured PDMS substrate has a nearly perfect cylindrical cap cross section, while the liquid alloy printed line showed a more irregular shape of the cross section. However, when deposited on a fully cured PDMS, the liquid alloy printing shows higher homogeneity over the length of a line although with severe roughness, Fig. 3 (a)–(d).

The discrepancy of the line width and the spacing between the designed patterns and the processed patterns comes from the cutting plotter performance, which also limits higher resolution^27^,^28^ (more detailed information can be found in Fig. S2 in the ESI). Depending on the atomization and spraying conditions, the 550–600 μm wide line of the liquid alloy coil had a height between 10 μm and 140 μm.

**Figure 4:**
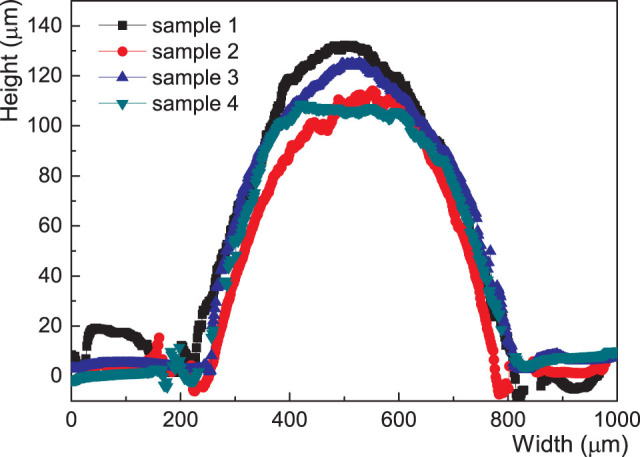
Morphology of liquid alloys. Morphologies of patterned lines of four different coils, which are fabricated under the same processing conditions.

The thickness of the sprayed pattern of the liquid alloy can, in part, be controlled by the spraying pressure, scanning speed and angle, frequency, time and finally the amount of the liquid alloy. The repeatability of the newly proposed process was checked by measuring the optical profile of the patterned line. The measured results indicated for thick layers a reasonable repeatability of ±20% in our current spraying process with manual operation, Fig. 4. It is anticipated to control the width of a liquid alloy pattern than its thickness in the current manual processing condition. The resistance of the fabricated coil for testing the efficiency of wireless power transfer was 8.1 Ω, which was of 600 μm width, 120 μm thickness and 82 cm length (Fig. S3 (a) in the ESI).

### Stretchable liquid alloy coil and integrated wireless power transfer device

**Figure 5:**
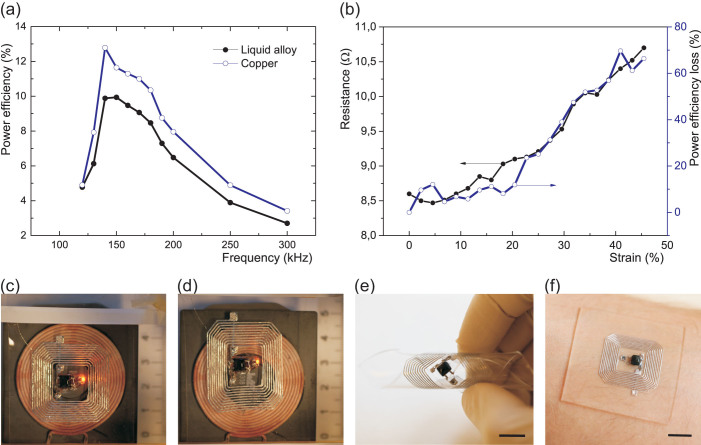
Performance of the liquid alloy coil for wireless power transfer. Resonant frequency vs. power efficiency of the patterned liquid alloy coil with a reference coil (a), resistance change related to power efficiency loss at applied strain and misalignment (b), and the fabricated wireless power transfer device with an LED for visualized working with different strains at 0% (c) and 25% (d), and in various states such as rolled state (e) and conformal to human arm (f), respectively.

The stretchable liquid alloy coil worked reliably before its breaking point over 50% strain with assembled copper wires connecting to instruments for testing of electrical properties through PDMS packaging, when no chip was integrated inside it. Figure 5 (a) shows the power efficiency of the liquid alloy coil that had 120 μm thickness in PDMS, while Fig. 5 (b) shows the resistance change related to power efficiency loss at applied strain and misalignment. We obtained the maximum power efficiency with the liquid alloy coil, which was approximately 10% corresponding to R_x_ power of 0.47 W at 140 kHz from the transmitting (T_x_) coil to the receiving (R_x_) coil when the T_x_ power was 4.6 W as shown in Fig. 5. The frequency response was not symmetrical around the tuned center frequency; at lower frequency region, the power efficiency dropped more rapidly compared to the higher frequency region. The reference copper coil had approximately five times lower resistance than the liquid alloy coil and the power efficiency of 13%. The performances of the coils are summarized in Tab. 1.

With the introduction of a hybrid-integrated circuit, Fig. 5 (c)–(d), in a localized stiff cell^14^,^15^, the stretchability of the sample was lowered to 25% from 50%, due to the stress concentrations at its interface with the stretchable surrounding. The microfluidic wireless power transfer device, with the R_x_ circuit which was hybrid-integrated with an LED was stretched to 25% of its initial length in one direction. The LED was lightened all the time but the lighting intensity was decreased due to the misalignment of the coils and the increase of the resistance of the liquid alloy coil, Fig. 5 (c)–(d). Mechanical cycling tests demonstrated that the device could survive more than 1,000 times of cycling between 0% and 25% strain done manually at 1 Hz, Fig. S4 in the ESI. After stretching tests, the liquid alloy line surface had stretch fissures, Fig. S5 in the ESI. In addition, although the stretchability was somehow reduced by introduction of rigid components, the integrated device can be still rolled, Fig. 5 (e), and conformally attached on human body such as arm skin, Fig. 5 (f).

### Verification of the process repeatability impact on the performance of the final fabricated coils

**Figure 6:**
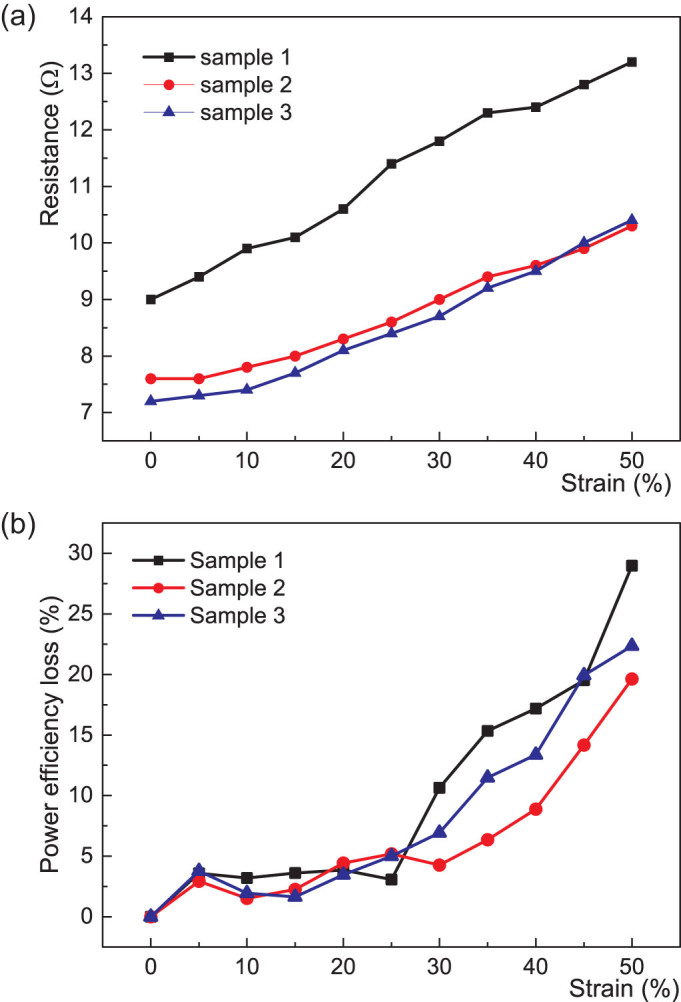
Resistance and power efficiency loss vs. strain. Measured resistance (a) and the corresponding power efficiency loss (b) of three different liquid alloy coils under strains, which are fabricated under the same processing conditions. The coils are increasingly misaligned due to the increased strain.

The DC resistance and the corresponding power efficiency loss under strain were measured for three fabricated coils, Fig. 6. The resistance of the liquid alloy coils was increased by strain, with the same behavior of the three samples. Power efficiency losses of the samples by strain showed similar behavior and they could work at high strains without losing much of the transferred power. The variation in resistance affects the power efficiency only to a small degree.

## Discussion

This tape transfer atomization patterning technique is very different from the previous liquid alloy printing technologies, which relied heavily on the wetting of liquid alloys to a substrate^15^,^16^. For instance, our liquid alloy printing technology was sensitive to the wetting of the liquid alloy on the substrate and the stencil mask as well as of the printing roller^21^, while another approach relied heavily on a thick metallic wetting layer, which might potentially change the composition of eutectic liquid alloys and hence their physical properties, such as the melting point^22^. Compared to our previously used liquid alloy printing technique, the tape transfer atomization patterning produced a line that has much better shape definition, as shown in the zoomed in photos, and edge shape when a semi-cured PDMS substrate was used, as shown in Fig. 3. This more uniform shape significantly increases the line conductance and makes it more predictable, in contrast to the large variation from the previous printing technique^21^.

The major reason for the improvement of the patterned shapes could be from better wetting of the liquid alloys onto the substrate by the high impact force when the atomized liquid alloy droplets hit the surface with their high density and high velocity during the spray deposition. Oxidized layers are naturally created on the surfaces of the droplets in air, which produces a reduced surface energy. This can help the liquid alloy to wet well on the substrate and not to spread out on the surrounding surface of the substrate when the mask is removed. Both aspects of high impact force and oxidized layers of liquid alloy droplets will improve patterning ability by increasing wettability of liquid alloys to the surface of the substrate. The stronger adhesion of a liquid alloy to the substrate helps the pattern to keep its shape better when the mask is removed, but the oxide layer of the liquid alloy also wets quite well to sidewall surface of the tape mask. Hence, a peeling-off speed of the tape mask below 1 mm/s was necessary to make a good pattern of the liquid alloy during the processing. Another reason for the improvement of the liquid alloy patterning with atomization is that the small sized droplets can precisely cover the open areas of the tape mask by following the pattern shape. In addition, the non-contacting coating process can help to make better shape and smooth surface morphology by merging the liquid alloy's droplets during deposition. All of these effects improve the patterning of the liquid alloy to the substrates.

This approach can also work on other substrates such as elastomers other than PDMS (e.g. polyurethane or other kinds of silicone based elastomer such as eco-flex), hard plastics (e.g. polymethylmethacrylate, PMMA), paper and even on substrates with very low surface energy such as Teflon (For more details, see Fig. S6 in the ESI). The quality varies with different substrates but it has a high potential to be further improved by tuning the process operation parameters.

Profilometry indicated reasonable repeatability in our current spraying process with manual operation when making power transfer coils, Fig. 4. However, this was with 100–140 μm thick layers and much thinner layers should have lower repeatability with their shorter timing. The height control should be possible to improve to ±5% by introducing dedicated, automated spraying equipment that can more precisely control the atomization process temporally and spatially. Nevertheless, the liquid alloy coil fabricated by this atomization patterning process has been shown to have significantly higher repeatability and reliability for wireless power transfer when a long line of a liquid alloy, which is nearly one meter long, on a soft substrate was patterned. As shown in Fig. 6, repeatability of the spraying process for liquid alloy patterning has been evaluated with optical profilometry and the similar behavior of resistance change by strain. The resistance variations of the samples could be from variations of cross section areas of the patterned liquid alloy coils and from the contact resistance of copper wires to the liquid alloy pattern, which was inserted for the measurement. In addition, although the oxide layer can effectively protect the liquid alloy for further oxidization in a static state and no change in resistance is seen after strain cycling (Fig. S4), long-term stress cycling is needed to further the understanding of stress induced oxidation.

**Figure 7:**
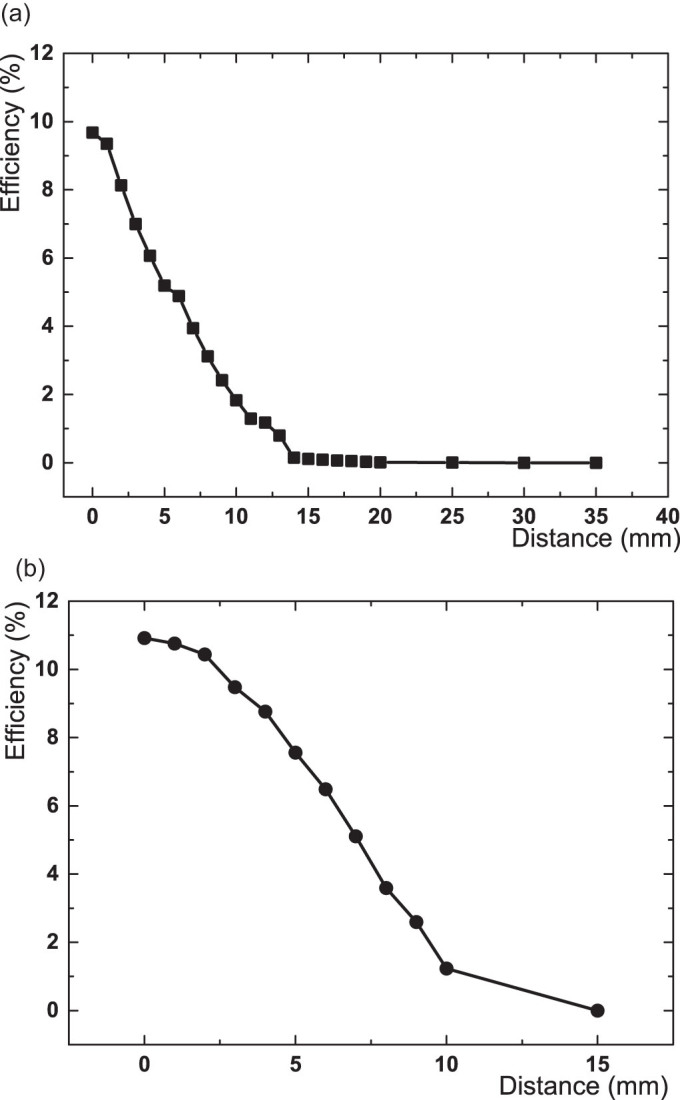
Power efficiency of the liquid alloy coil dependent on distances. Power efficiency changes of a liquid alloy coil by increasing the distance apart from T_x_ coil's top surface to R_x_ coil (a), and by increasing the distance of off-axis shifting from the centre point of R_x_ coil to the centre point of T_x_ coil (b).

**Figure 8:**
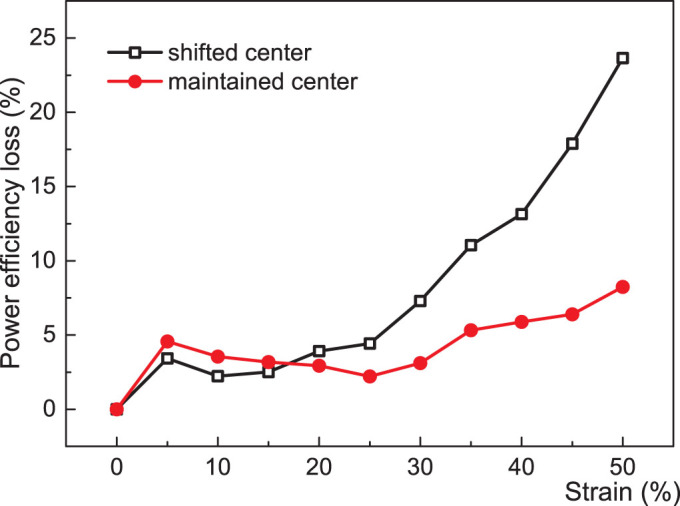
Comparison of the power efficiency loss with the increasing strains when the center is shifted from or maintained at the co-axial center position.

In the test of wireless power transfer, the power efficiency change was tested by the distance between the planar surfaces of the two coils in Fig. 7. Transferred power change was measured when the R_x_ coil was moving out of the coaxial position. When it was misaligned by 15 mm from the coaxial center point, the transferred power was diminished, Fig. 7. And we found that the power transfer efficiency was reduced by strain, which was partially due to the resistance increase by stretching, but an induced misalignment when stretching affected the efficiency loss more than the resistance change by strain did, Fig. 8. To implement the microfluidic wireless power transfer device with an LED indicator, a shifted center alignment between the transmitting and receiving coils was used for better visualization of the stretchability. However, when a center alignment mode was used, the power efficiency was higher during the stretching, and even the variation of power efficiency loss was decreased along strain increase.

The main limitation in the stretchability of the device in our test lies in the large footprint of the rectifier on the flexible printed circuit board inside the PDMS encapsulation. To further enhance its stretchability, it could be potentially improved by using a bare die of a rectifier chip or an RF chip. There are several factors to consider if a stretchable coil is to be optimized for a specific application. Optimization of the total efficiency of a wireless power transfer system of a liquid alloy based coil would be possible with fine tuning of the coil's properties by changing the geometry, including height, width and spacing of each turns of the coil line, and also by increasing numbers of turns or stacking of several coils^29^. The coupling factor (k factor) could be increased with higher electromagnetic coupling by a use of ferrite cores. Resonant frequency matching between T_x_ and R_x_ should be made by a low frequency network analyzer, considering impedance matching of the circuits, simultaneously.

In conclusion, this work demonstrated a versatile liquid alloy patterning technique on soft substrates based on tape transferred adhesive masking, and atomization deposition of a liquid alloy. It proved that this technique is able to pattern high quality, uniform and clear patterns of 500 μm wide lines with lengths near a meter. A microfluidic wireless power transfer device was successfully demonstrated with excellent mechanical stretchability as well as good power transfer performance. By introducing a multilayer fabrication technique, an integrated wireless power transformer with an LED was demonstrated. Further improvement and optimization of this process technique could offer higher resolution and repeatability, and create new opportunities to introduce advanced functions in conformal devices, intelligent systems on the skin or what could be implanted for man-machine communication.

## Methods

### Patterning the liquid alloys on a semi-cured PDMS

The PDMS substrate was prepared by mixing with 9:1 weight ratio of the base and cross linker (Elastosil RT601A and B, Wacker Chemie, Munich, Germany) and degassing trapped air bubbles in a refrigerator at −20°C. After that, PDMS was spun onto a 4-inch silicon substrate at 1,100 rpm. The spun PDMS substrate was semi-cured at 70°C on a hot plate. In parallel, the shadow mask for liquid alloy patterning was prepared with a vinyl tape on a wax-coated paper liner (L & M series, RITRAMA, Italy) by a commercial signage cutting plotter (CraftRoboPro, Graphtec, Japan). Subsequently, the undesired parts were removed after the cutting. The prepared mask was then transferred onto a semi-cured PDMS substrate with a transfer tape (ApliTape 4050, Rtape Corp., USA). After the removal of the transfer tape from the mask pattern adhered on the PDMS substrate, atomization deposition of liquid alloys was done with a manual art airbrush (Meec tools, Jula, Sweden) coupled to a pressure regulator (SL101–220, I&J Fisnar, USA), following via-hole punching with a circular shaped knife for multilayer structuring of the liquid alloy circuit. A volume of 1.5 ml of the liquid alloy was used for spraying over the masked area of a coil, 20 × 20 mm^2^, and the pressure with nitrogen gas was set 30 psi at the pressure regulator. Finally, the liquid alloy pattern was obtained by removing the vinyl adhesive mask from the PDMS substrate.

For comparison, our recent liquid printing technique was also used with tape transferred cut adhesive masks by adapting the protocol in the previous work^21^. The patterning was evaluated by optical microscopy and profilometry, studying the liquid alloy line widths from 200 μm to 500 μm with the fixed spacing of 500 μm between the lines and spacing variations from 200 μm to 500 μm with the fixed line width of 500 μm.

### Rigid circuit embedment and multilayer fabrication

To implement the microfluidic wireless power transfer device, a multilayer technique was developed and the R_x_ circuit was hybrid-integrated with an LED. The LED was used as an integrated load as well as an indicator of the wireless power transfer system, Figure 5 (c)–(f).

Depending on the application, the fabricated liquid alloy patterns on a PDMS substrate could be encapsulated with another layer of PDMS or integrated not only with passive or active components but also with circuitry, and even stacked with multiple layers (Figure S1 in the ESI). Flexible PCB, a copper layer on polyimide film, was etched with FeCl_3_ (Elfa, Sweden) solution to assemble with the liquid alloy coil for the R_x_ part. Several rigid chips, including a diode, a resistor, capacitors and an LED, were soldered onto the flexible PCB with a silver paste (CR 44 SMD Lotpaste, Farnell, Sweden) in a reflow oven (ProtoFlow, LPKF, Germany). After cutting the proper shape of flexible PCB, it was integrated with the liquid alloy coil. The assembled device was encapsulated with another layer of PDMS. To pattern a second layer of the liquid alloy circuit on the back-side of the PDMS substrate after finishing the front-side encapsulation, the same process as the first circuit layer was repeated, with a different mask on the opposite surface of the PDMS substrate, by aligning with via-holes prepared before spraying the first circuit layer. Finally, the second layer of the liquid alloy circuit, with integration of chips if needed, was encapsulated with a third layer of PDMS. For testing the stretchability and efficiency of the wireless power transfer system, copper wires were placed on liquid alloy pads to connect measurement instruments after encapsulating with another layer of PDMS.

### Processing comparison on various substrates

Besides the PDMS substrate that we normally use, liquid alloys can be patterned onto other soft material based substrates (silicone, EcoFlex 0030, Smooth-on. Inc, USA; Poly(methyl methacrylate), PMMA, ME303016, GoodFellow, UK; polyimide, TL Series Polyimide, Saint Gobain, France; polycarbonate, PC, Makrolon mono, Bayer Material Science, Germany; Polyurethane, PU, 100 μm Sheet, BASF, Germany; Polyethylene terephthalate, PET, Panaprotect CT100, Panac, Japan; Polytetrafluoroethylene, PTFE, Dupont Teflon FEP, Dupont, USA; 200 g premium A4 Paper from a local stationary store) (Fig. S6 in the ESI). This was once again compared with our recent liquid printing technique^21^.

### Optical characterization

An optical microscope (Provis AX70, Olympus, Japan) was used for observation of the liquid alloy patterns and an optical profiler (Wyko NT1100, Veeco, USA) was used to measure the height and roughness of the liquid alloy patterns.

### Coil and circuit design

The coil size was designed with the outer dimension of 30 mm × 30 mm and the inner dimension of 13.5 mm × 13.5 mm. As a reference, a same sized 35 μm thick copper coil on 1.5 mm thick FR4 board was also made, Fig. S3 (b) in the ESI.

### Mechanical measurement

The stretchable devices were manually stretched, being fixed in a slider made of hard plastic. The stretching was clocked and made with a frequency of 1 Hz for 1,000 times in a row before checking the electrical performance. The manual test setup is shown in Fig. S7 in the ESI.

### Electrical measurement

The total efficiency of the wireless power transfer system is defined as:
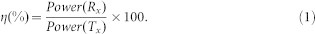
 The power efficiency loss, η_loss_ in Eq. 2, was calculated from the measured power efficiency without strain, η_i_, and the power efficiency with strain, η_s_, when stretching the coil: 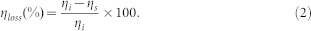
 The R_x_ coil was laid on the top of the T_x_ coil by direct contact, with only a PDMS substrate thickness of 100 μm between them. The transmitting power was read from the power supply that was applying power to the transmitting (T_x_) circuit. Received power was calculated by measuring the voltage and current in the receiving (R_x_) circuit after rectification. The impedance matching of the R_x_ circuit was done by changing its resistance. For the fabricated liquid alloy coil, an impedance matching resistance of 107 Ω showed the maximum power transfer, Fig. S8 in the ESI. The designs of the T_x_ and R_x_ circuit are shown in Fig. S9 in the ESI.

During the electrical test of the wireless power transfer system, a power supply (QL355P, TTi, UK) and a function generator (33120A, Agilent technologies, USA) were connected to the T_x_ circuit. Multimeters (34450A, Agilent technologies, USA) were used to measure the voltage and current from the R_x_ part. During the stretching tests, the distance from the top surface of the T_x_ coil to the bottom surface of the R_x_ coil was 5 mm and one side of the R_x_ coil was fixed at its initial position.

## Author Contributions

Z.W. and S.J. designed the work. S.J. carried out the experimental work. S.J., K.H. and Z.W. contributed to the data analyzing and manuscript writing. Z.W. initiated and supervised the work.

## Supplementary Material

Supplementary Information Supporting Information to Tape Transfer Atomization Patterning of Liquid Alloys for Microfluidic Stretchable Wireless Power Transfer

## Figures and Tables

**Table 1:** Comparison of the power efficiencies. Power efficiencies of a copper- and a liquid-alloy-based power transfer device

	Sample resistance	Freq-uency	T_x_			R_x_			Power efficiency
			Voltage	Current	Power	Voltage	Current	Power	
	Ω	kHz	V	mA	W	V	mA	W	%
Copper	1.6	140	20	200	4.0	7.4	70	0.52	13
Liquid alloy	8.1	140	20	230	4.6	7.0	68	0.47	10
