# Supplementary material for: Tape Transfer Atomization Patterning of Liquid Alloys for Microfluidic Stretchable Wireless Power Transfer
Source: Sci Rep. 2015 Feb 12;5:8419. doi: 10.1038/srep08419 (PMC4325334; doi:10.1038/srep08419)
Supplement: Supplementary Information — Supporting Information to Tape Transfer Atomization Patterning of Liquid Alloys for Microfluidic Stretchable Wireless Power Transfer [file srep08419-s1.doc]

Supplementary Information

Tape Transfer Atomization Patterning of liquid alloys for Microfluidic Stretchable Wireless Power Transfer

Seung Hee Jeong^1^, Klas Hjort^1^, and Zhigang Wu^1,2^*[[1]](#footnote-2)*

^1^Department of Engineering Sciences, The Angstrom Laboratory, Uppsala University, Box 534, 75121, Uppsala, Sweden

^2^State Key Laboratory of Digital Manufacturing Equipment and Technology, Huangzhong University of Science and Technology, 430074, Wuhan, China

R_x_ circuit design

The resonance frequency of the T_x_ coil was calculated by the measured resistance, inductance of the T_x_ coil and the circuit impedance load. For higher power transfer efficiency, the impedance of the T_x_ and R_x_ parts should be matched with each other to make a resonance frequency matching by tuning capacitance in the circuit. The resonance frequency was calculated with the impedance of the T_x_ part by

$f_{res}=\frac{1}{2}\pi\sqrt{L\times C}$ (S1)

where 𝑓_res_ is the resonance frequency, L is the inductance, and C is the capacitance of the coil. The circuit diagrams of T_x_ and R_x_ circuit are shown in Fig. S10. The inductance of the transmitting coil and C1 in the circuit was set as 24 μH and 200 nF, respectively, which allowed C2 to be calculated after measurement of L2, using the same equation. The inductance of the copper coil and the liquid alloy coil was set to be the same, at 4 μH, and the inductance of transmitting coil (WÜRTH elektronik, Germany) was 24 μH as measured by an LCR meter (Precision LCR meter 4284A, Hewlett-Packard, USA) at 100 kHz with a series connected inductance and resistance model. The size of the embedded circuitry was of 1 cm by 2 cm. The size of the coil was decided by following the recommendation of the wireless power transfer consortium^R1^, which is that the R_x_ coil diameter should be larger than half of the T_x_ coil diameter but smaller than the T_x_ coil diameter.

The quality factor (Q factor) indicates that the damping of frequency oscillations in a coil can also indicate the coupling efficiency of the system, and is dependent on inductance and resistance of the coil at the operating frequency. The Q factor is defined as

$Q=\frac{\omega L}{R}$ (S2)

where ω = 2π𝑓_res_ and R is resistance of a coil, respectively. When the operating frequency was 140 kHz, i.e. the designed resonance frequency of the system, the inductance was 4 μH and resistance was 8.1 Ω, The Q factor of the liquid alloy coil was calculated to be 0.4 and the Q factor of the T_x_ coil was 45, according to the datasheet provided by the manufacturer. A high electrical conductivity of the liquid alloy is important to make a high efficient energy transfer.

In electrodynamics theory, the optimal efficiency is given by

$\eta_{opt}=\frac{U^{2}}{(1+\sqrt{1+U^{2}})^{2}}$ (S3)

where figure of merit, U=$k\times\sqrt{Q_{1}\times Q_{2}}$, k is the magnetic coupling factor between the T_x_ and

R_x_ coils and Q is the quality factor of each coil. If the coupling factor was 0.5 as a practical assumption, which means all magnetic flux penetrates the receiving coil, the U is 2.12 and then the efficiency was calculated as 40%. Compared to the measured efficiency, which was 10%, as shown in Tab. 1, the calculated efficiency is much higher than the tested one. With the same arguments as above for the reference copper coil, with a U of 4.97, the efficiency is calculated to be 67% as compared to the measured 13%. There are several potential reasons for this, e.g., the efficiency loss that could be caused by the permittivity and permeability of the used substrate (100 μm thick PDMS or 1.5 mm thick FR4 substrate) of the R_x_ coil as well as the coupling factor dependency including co-axial aligned distance, coil to coil distance and facing angle. In the T_x_ and R_x_ circuits, a MOSFET (100 V, 36 A, N, International rectifier, USA), a bridge rectifier (200 V, 500 mA, Fairchild semiconductor international, USA), capacitors and resistors were mounted on a flexible PCB.

**Strain test and stretching cycling test**

For the strain test, the prepared liquid alloy coil in the PDMS packaging was connected to the circuit of the receiving part through copper wires inserted into it. The liquid alloy coil was stretched gently to observe changes of resistance, voltage and current by strain in the customized stretching frame which held the sample, with screws pressing on each end of the sample. For a cycling test, the same testing jig was used by manual stretching.

R1. URL http://www.wirelesspowerconsortium.com/technology/basic-principle-of-inductive power-transmission.html (accessed on 14 July 2014).


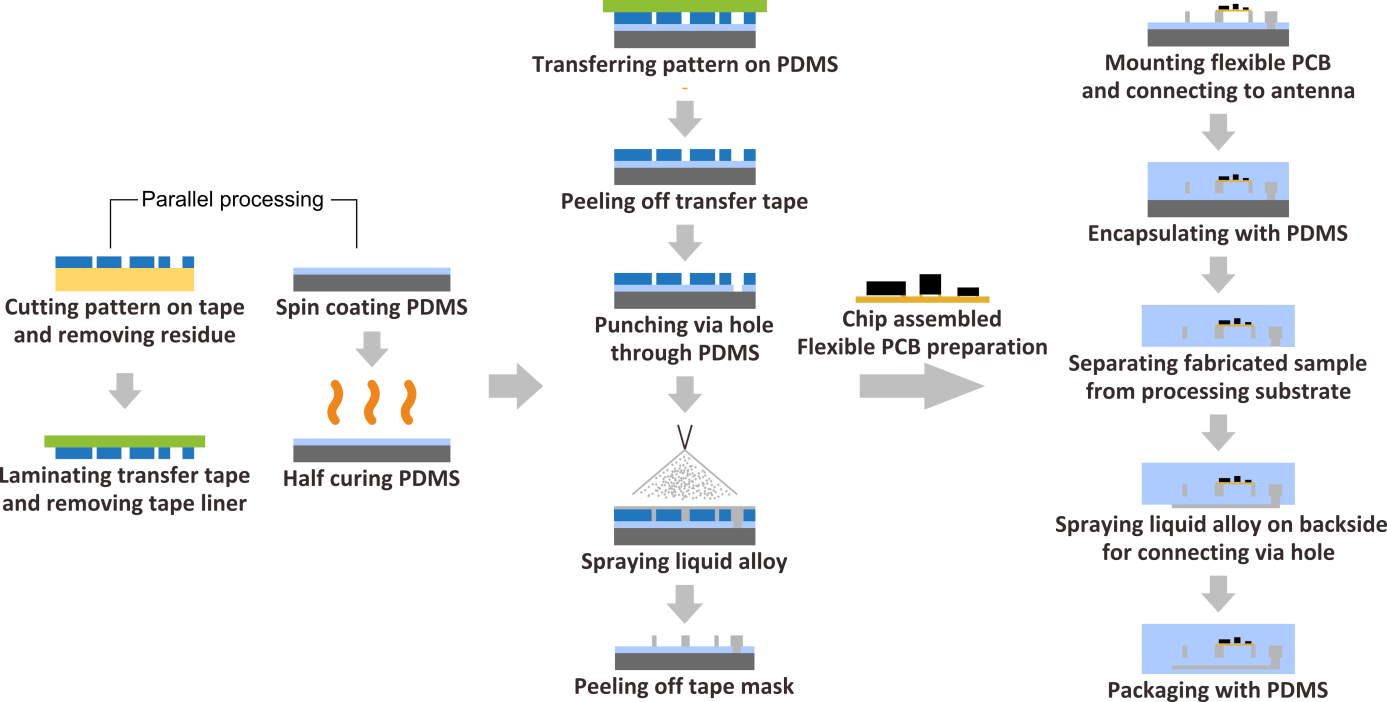


**Figure S1.** Fabrication process of the integrated stretchable wireless power transfer device.

**Figure S2.** Pattern size calibration of the line width and the spacing of the tape transfer process and atomization patterning of the liquid alloy with (a) different spacing distances between lines, and (b) different line widths.


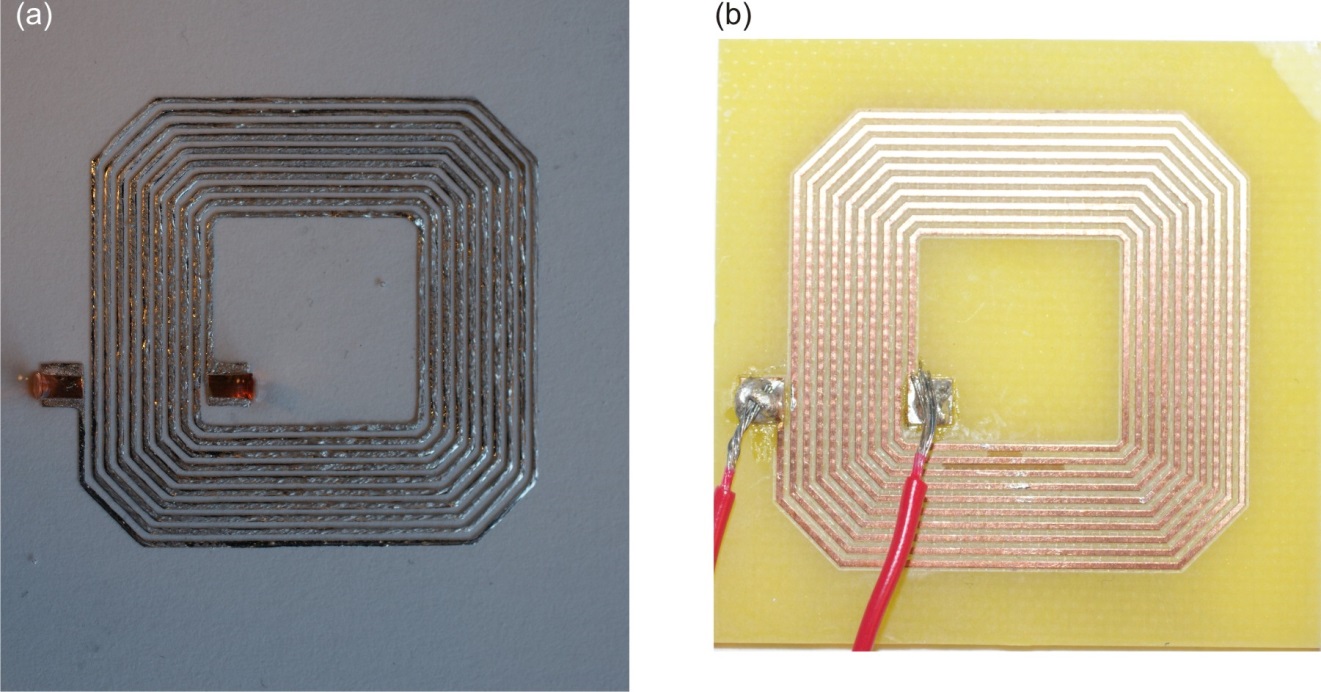


**Figure S3.** Photos of the microfluidic coil made of liquid alloy on PDMS (a) and the reference coil made of copper on FR4 (b).

**Figure S4.** Comparison of the measured resistances of three different liquid alloy coils (shown in Fig. 6) before and after 1,000 times cycling test under the strains from 0% to 25%. (The measurement contains both resistances when stretched and released.)


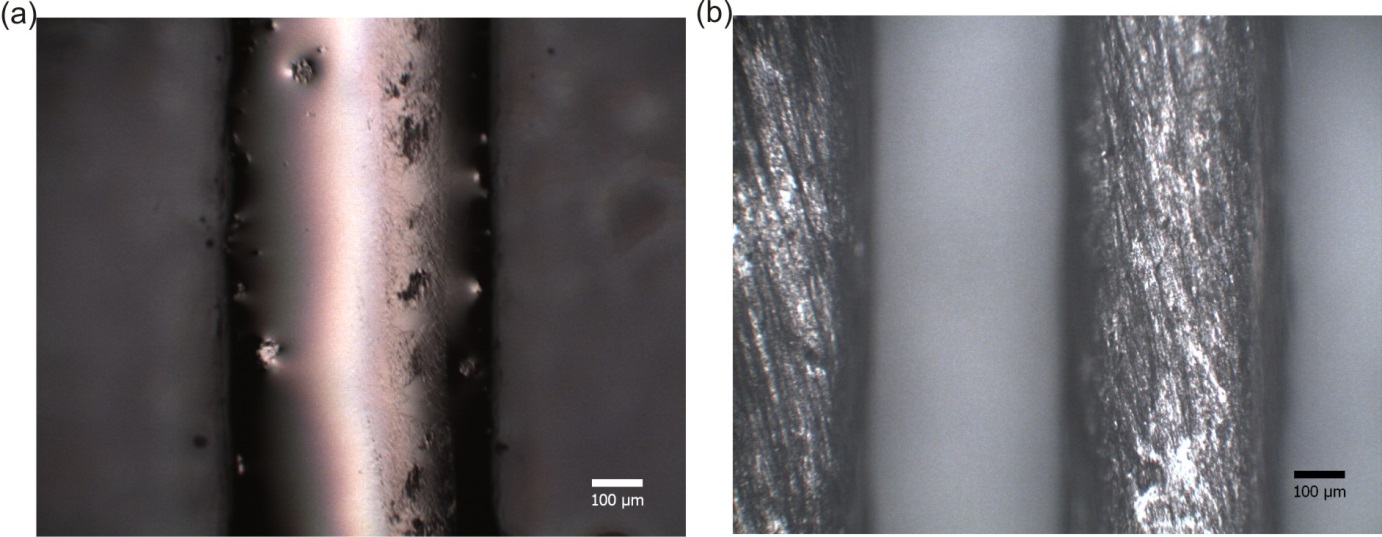


**Figure S5.** Micrographs of an atomized pattern of the liquid alloy before stretching on PDMS with a support of a silicon wafer (a) and after stretch cycling test encapsulated in PDMS without a rigid support (b).


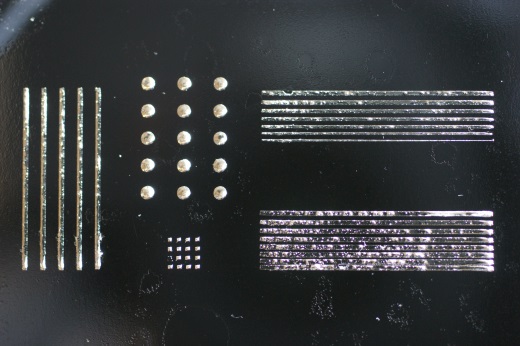

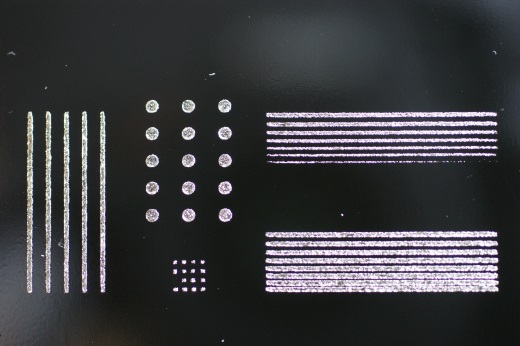


Elastosil (semi-cured)


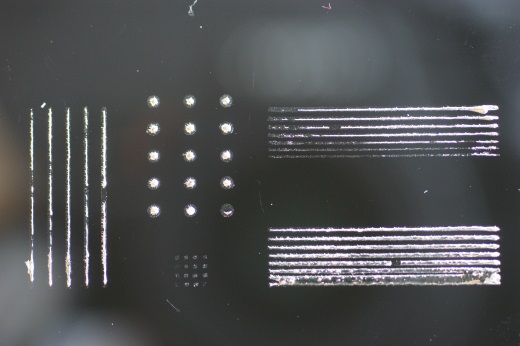

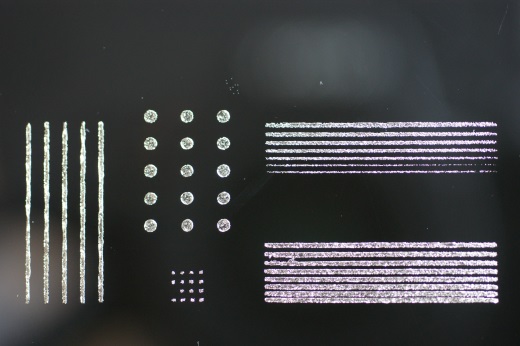


Elastosil (full cured)


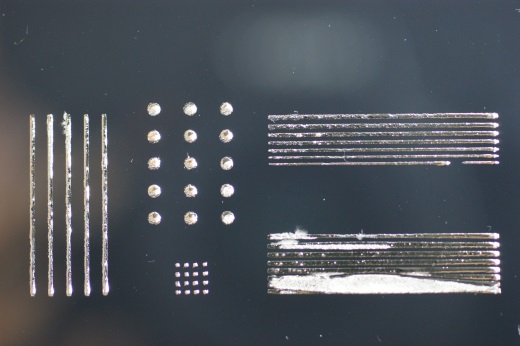

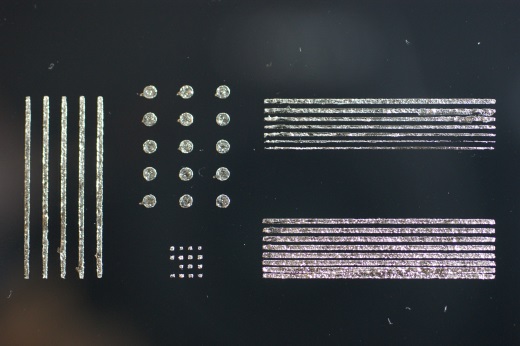


EcoFlex (semi-cured)


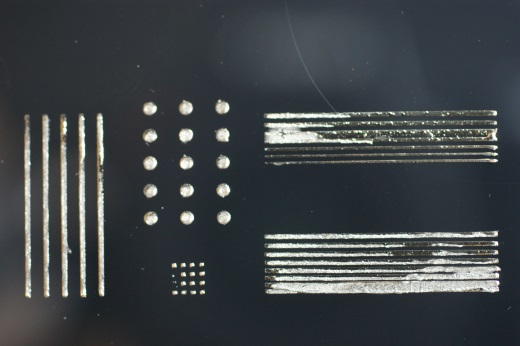

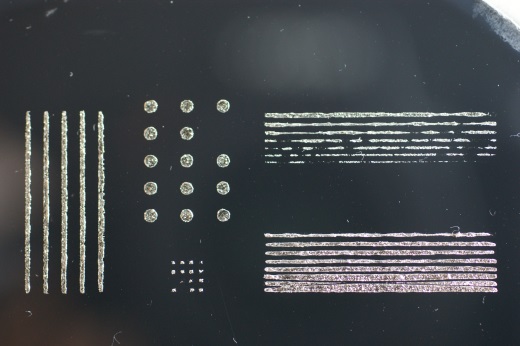


EcoFlex (full cured)


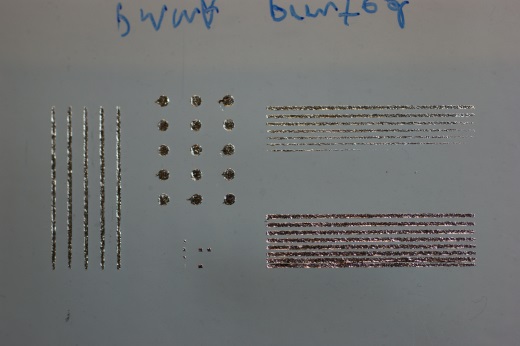

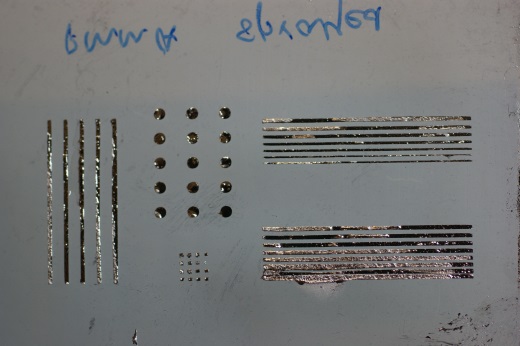


PMMA


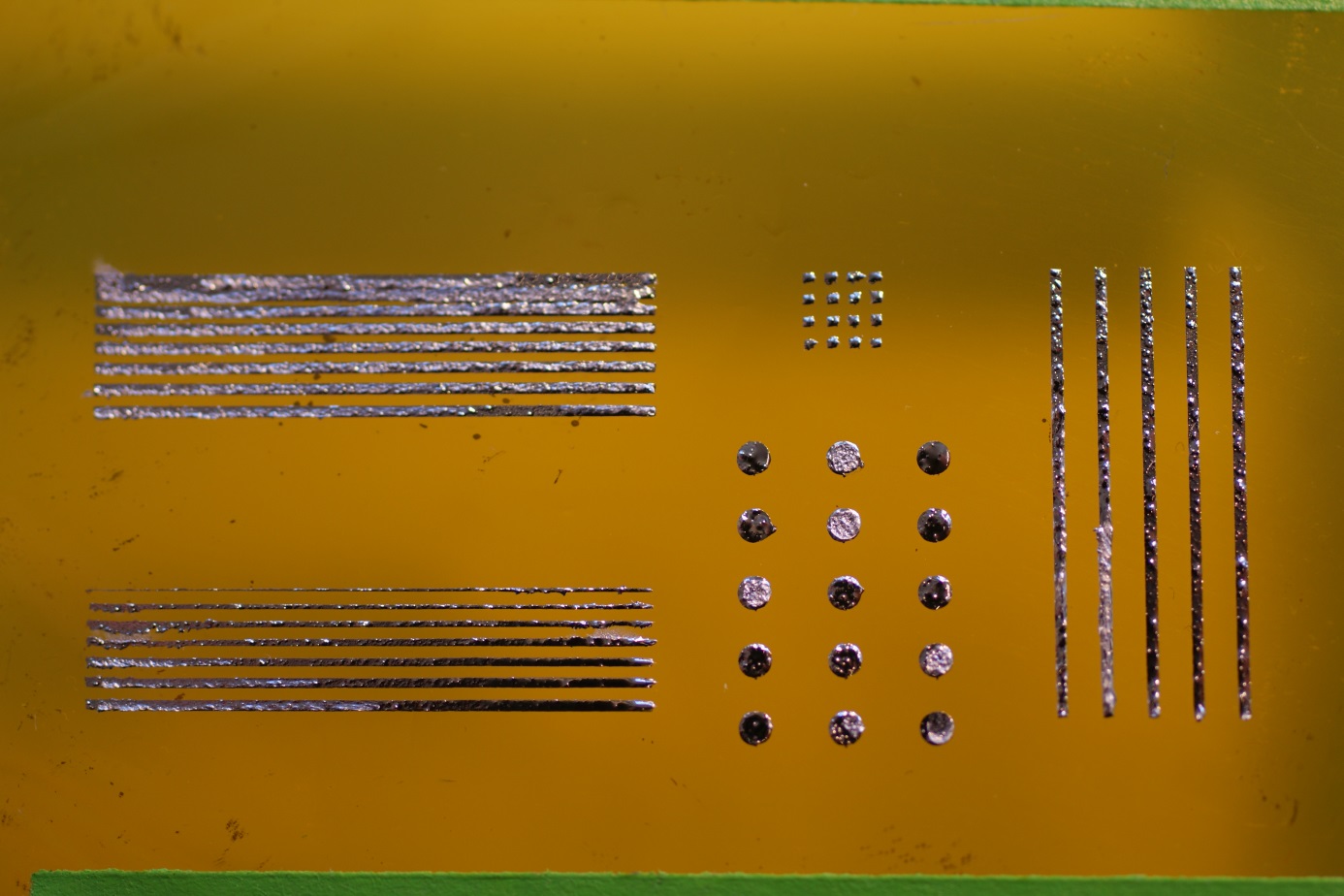

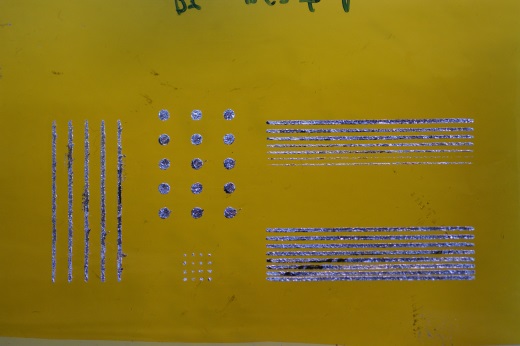


Polyimide


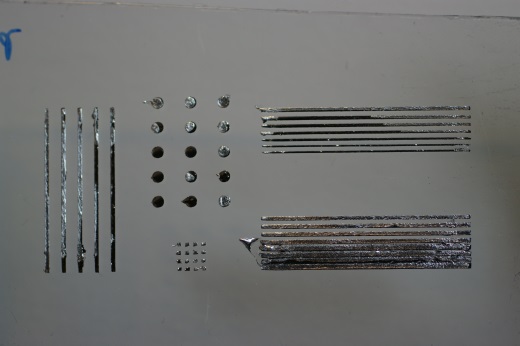

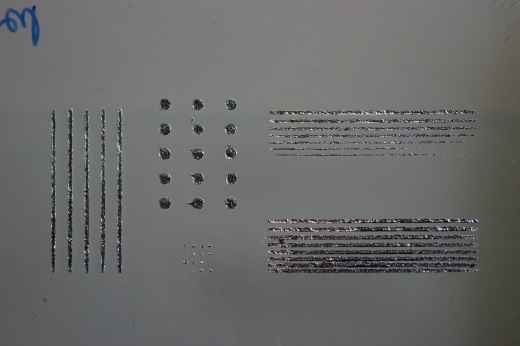


Polycarbonate


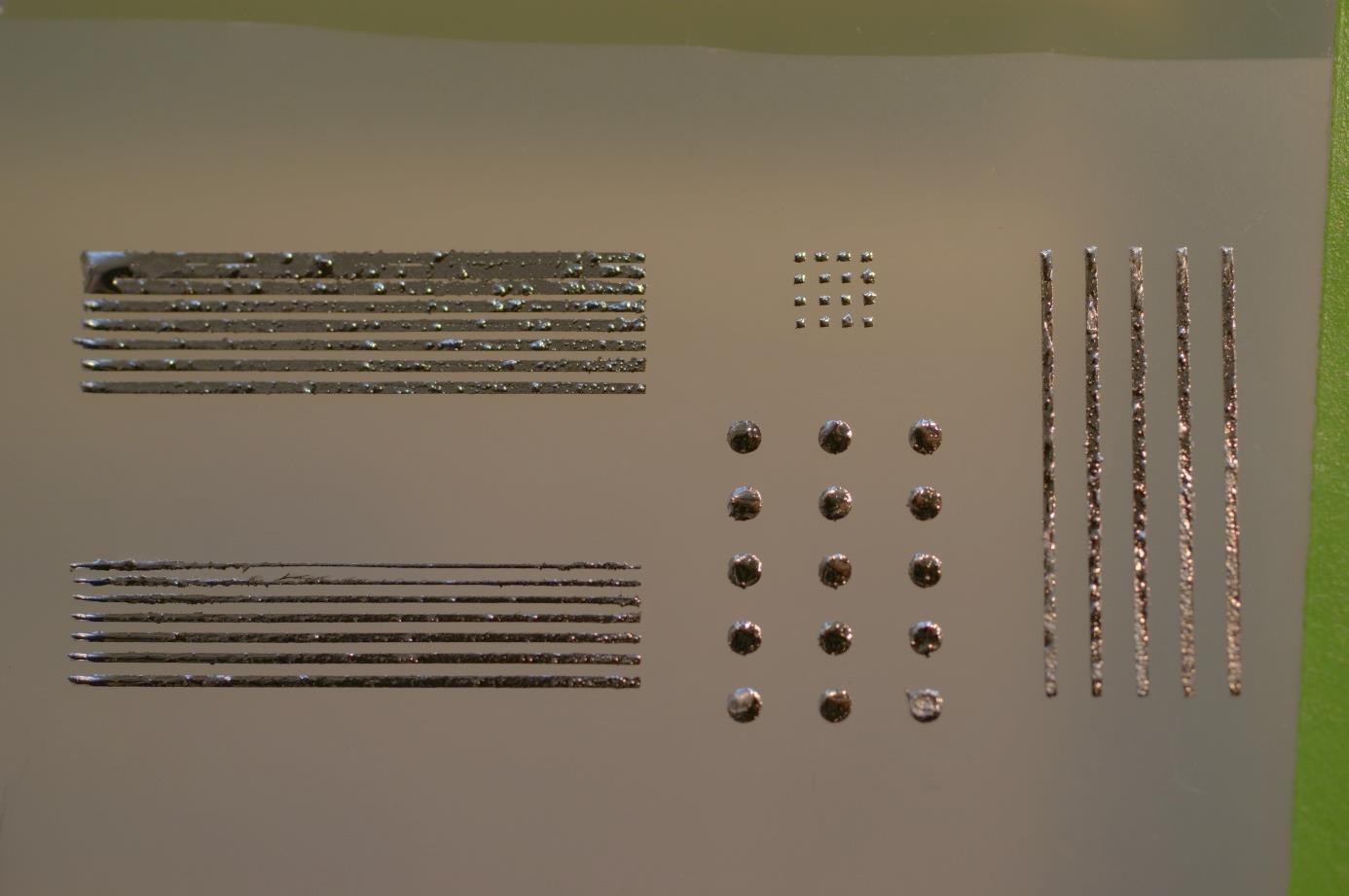

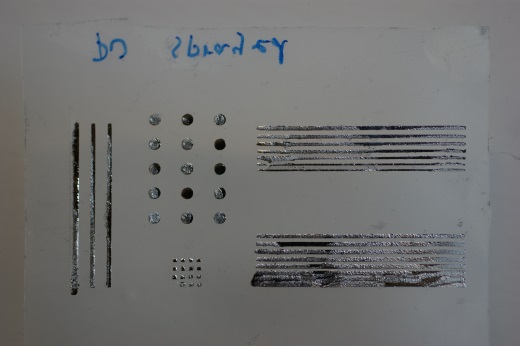


Polyurethane


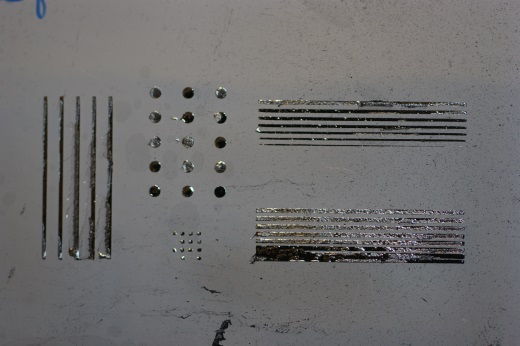

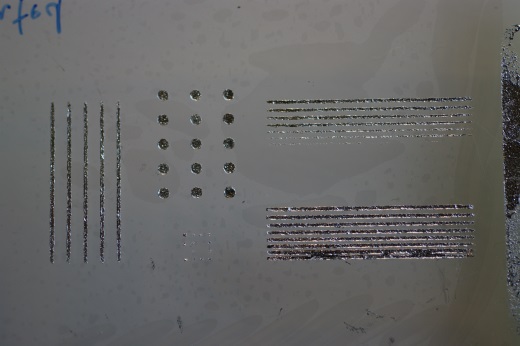


Polyethylene terephthalate


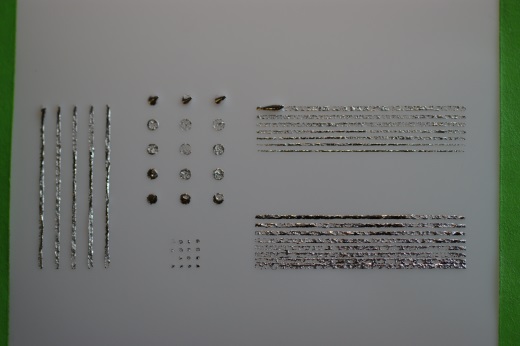

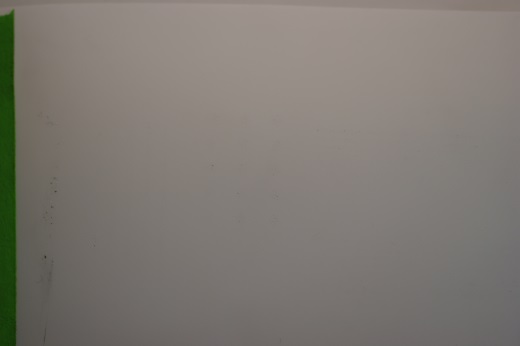


Teflon


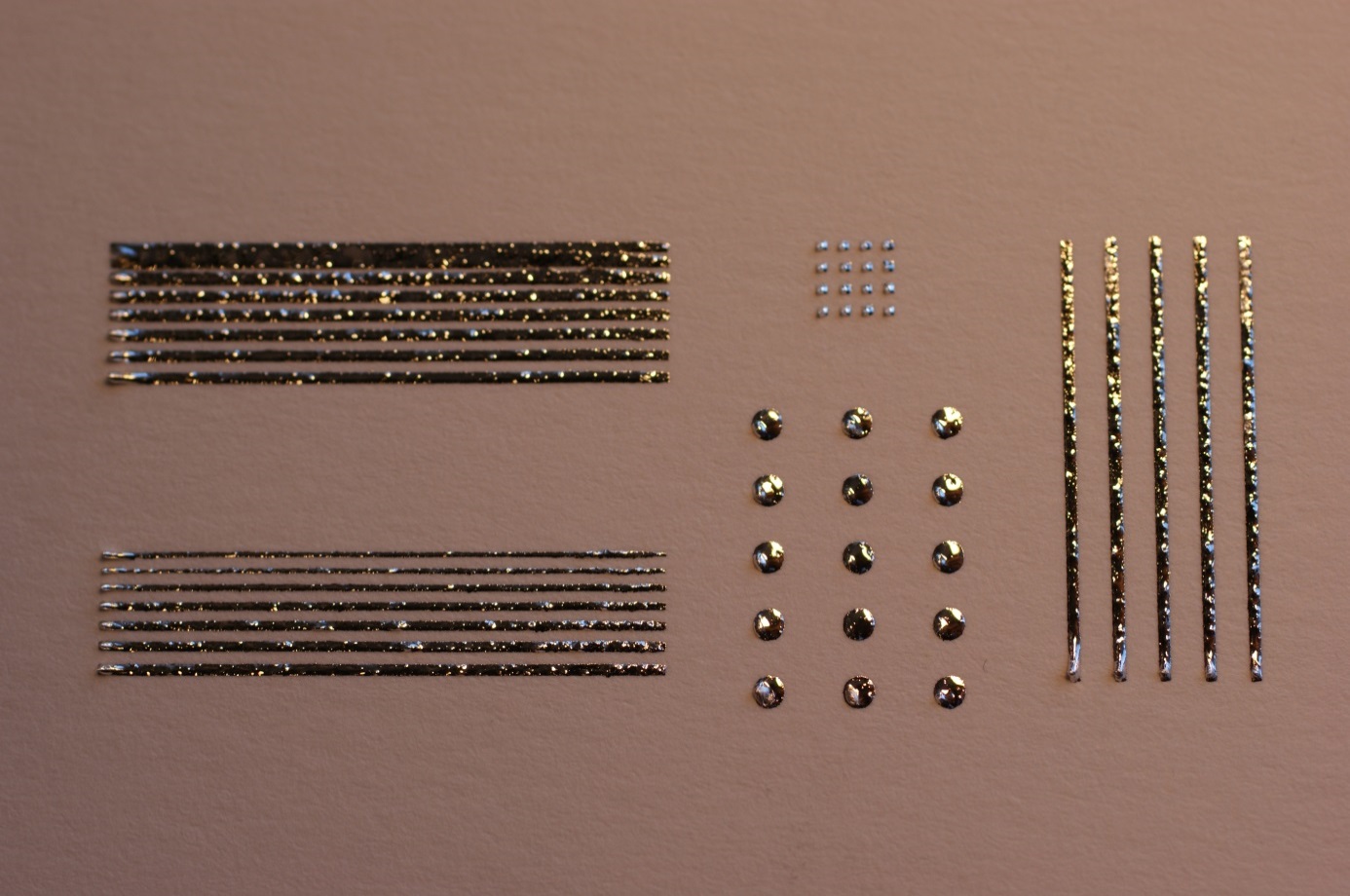

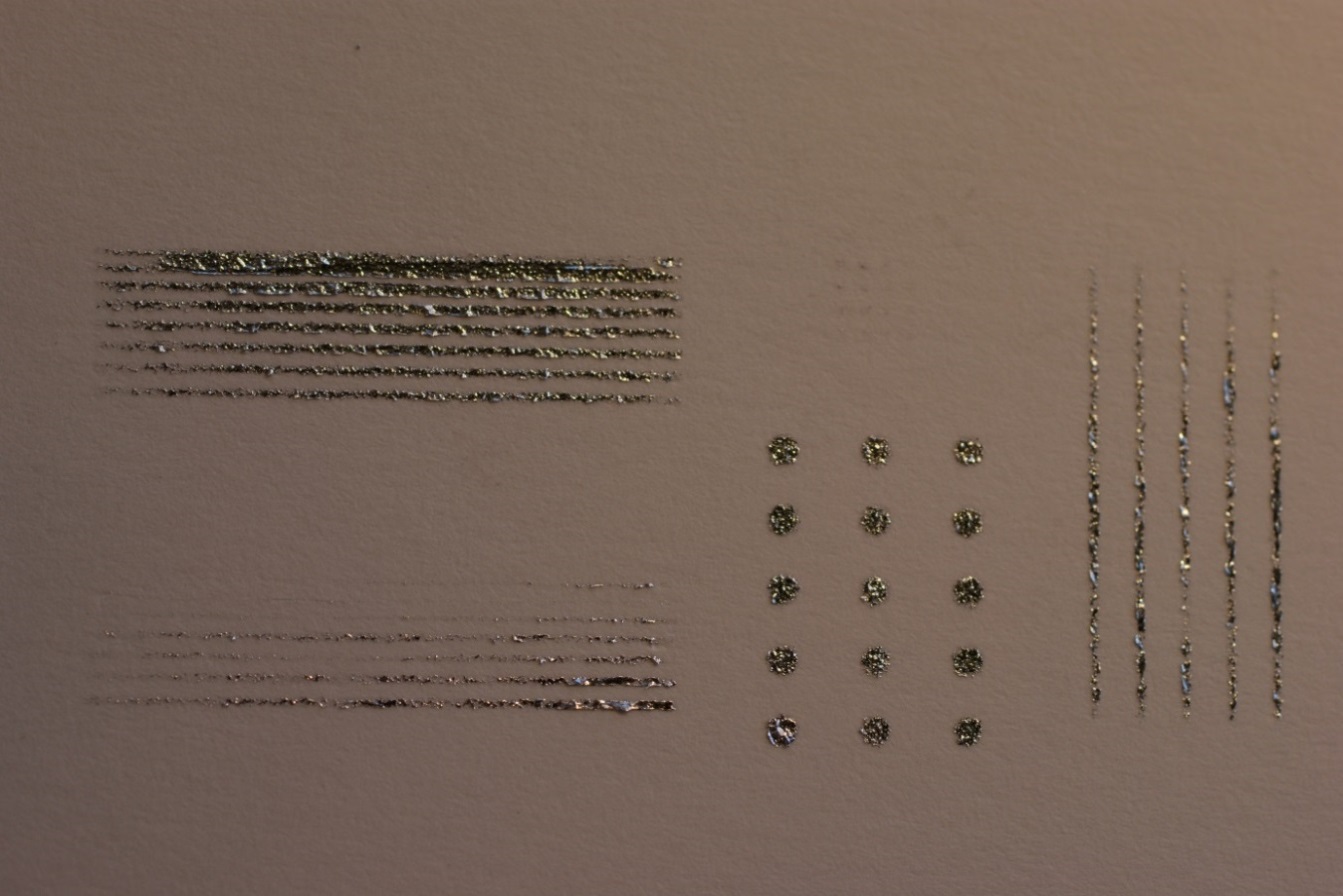


Paper

**Figure S6.** Patterned liquid alloys on the different substrates by atomization patterning (left)

and by roller printing (right) for each material.


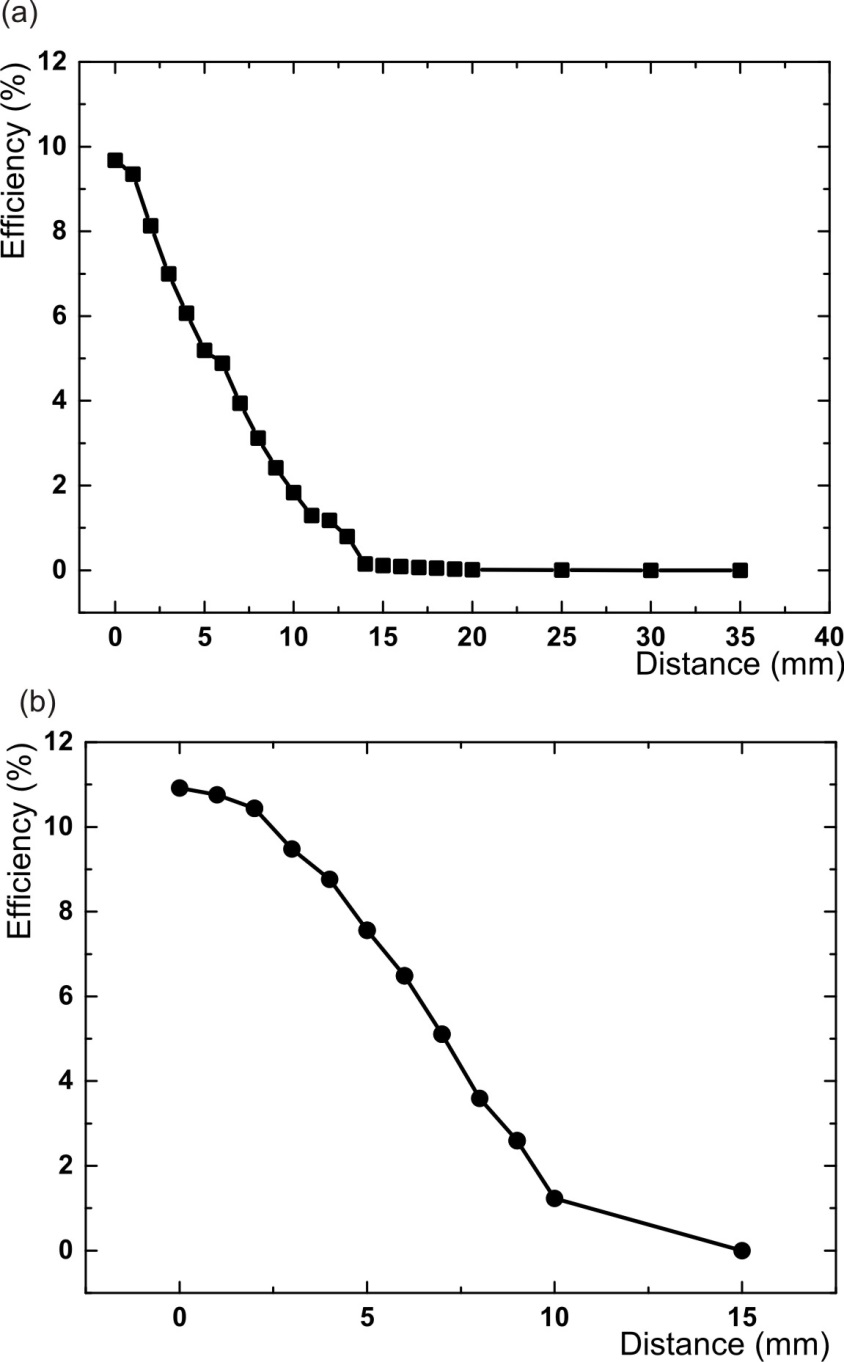


**Figure S7. Power efficiency of the liquid alloy coil dependence of distance.** Power efficiency changes of a liquid alloy coil by increasing the distance apart from T_x_ coil’s top surface to R_x_ coil (a), and by increasing the distance of off-axis shifting from the centre point of R_x_ coil to the centre point of T_x_ coil (b).

**Figure S8.** Comparison of the power efficiency loss with the increasing strains when the center is shifted from or maintained at the co-axial centre position.

**
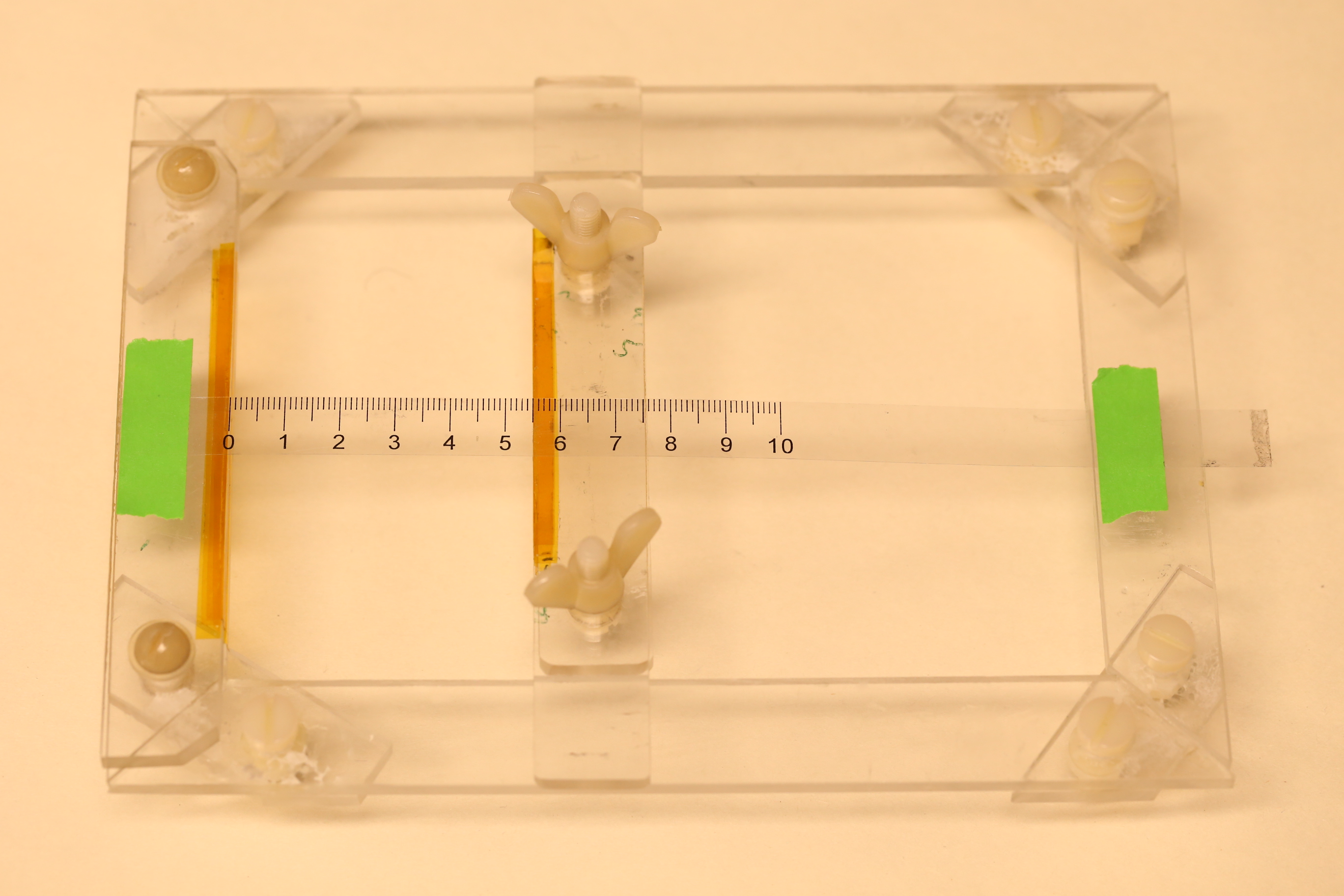
**

**Figure S9.** Photograph of the manual, mechanical, stretching test setup of the microfluidic liquid alloy coil on PDMS.


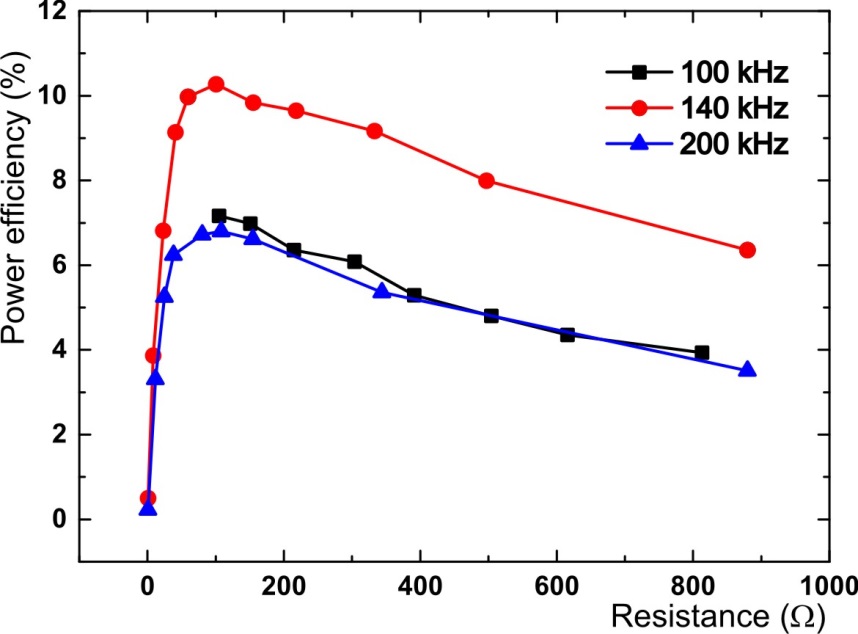


**Figure S10.** Power efficiency by impedance changes in R_x_ circuit with different frequencies.


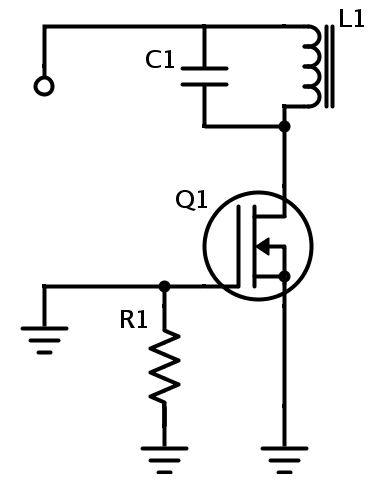


(a) Transmitter part


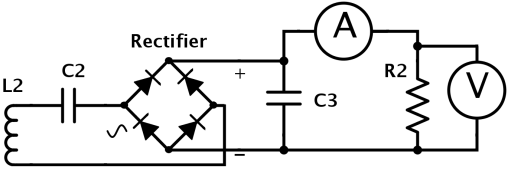


(b) Receiver part

**Figure S11.** Circuit diagrams of T_x_ and R_x_ circuit.

1. **Correspondence and requests for materials should be addressed to** [**Zhigang.Wu@angstrom.uu.se**](mailto:Zhigang.Wu@angstrom.uu.se)**. Tel: +46 18 471 1086.** [↑](#footnote-ref-2)
